# Three-dimensionally Ordered Macroporous Structure Enabled Nanothermite Membrane of Mn_2_O_3_/Al

**DOI:** 10.1038/srep22588

**Published:** 2016-03-03

**Authors:** Guoqiang Zheng, Wenchao Zhang, Ruiqi Shen, Jiahai Ye, Zhichun Qin, Yimin Chao

**Affiliations:** 1 School of Chemical Engineering, Nanjing University of Science and Technology, Nanjing, 210094, China; 2 School of Chemistry, University of East Anglia, Norwich, NR47TJ, United Kingdom

## Abstract

Mn_2_O_3_ has been selected to realize nanothermite membrane for the first time in the literature. Mn_2_O_3_/Al nanothermite has been synthesized by magnetron sputtering a layer of Al film onto three-dimensionally ordered macroporous (3DOM) Mn_2_O_3_ skeleton. The energy release is significantly enhanced owing to the unusual 3DOM structure, which ensures Al and Mn_2_O_3_ to integrate compactly in nanoscale and greatly increase effective contact area. The morphology and DSC curve of the nanothermite membrane have been investigated at various aluminizing times. At the optimized aluminizing time of 30 min, energy release reaches a maximum of 2.09 kJ∙g^−1^, where the Al layer thickness plays a decisive role in the total energy release. This method possesses advantages of high compatibility with MEMS and can be applied to other nanothermite systems easily, which will make great contribution to little-known nanothermite research.

In recent years, the demand for miniaturization and integration of power generator has significantly increased in microelectromechanical system (MEMS). Although chemical battery technologies have been greatly developed, the energy density is still relatively low. It is impossible to attain large energy release from the micro-scale batteries^1^. It is critical to find a new class of highly efficient materials for MEMS. Nanoenergetic materials (nEMs) are supposed to be one key to the great advance in power generator owing to their high energy density, high compatibility, and the improved microfabrication processes such as photolithography, thin film deposition and doping^2^,^3^.

Nanothermite, also termed metastable intermolecular composites (MIC), is a typical class of nEMs. An MIC generally consists of fuel (such as Al, Mg) and a variety of metal oxides (e.g. CuO, Fe_2_O_3_, MoO_3_, Bi_2_O_3_, NiO). Under the stimulus of flame, laser pulse, current or heating, an oxidation-reduction reaction breaks out and releases a large amount of energy. Owing to shorter mass transfer distance and larger contact area, MICs show better performance in energy release, ignition delay time and reaction rate compared to their bulk or micro counterparts^3^,^4^,^5^,^6^,^7^, and have also revealed promising applications in many fields including microinitiators, gas generators, microheaters and microthrusters^8^,^9^,^10^,^11^,^12^,^13^. In the past two decades, various techniques have been developed to prepare MICs, such as physical mixing^14^,^15^,^16^, sol-gel/aero-gel^17^,^18^, molecular self-assembly^19^,^20^,^21^, physical vapor deposition (PVD)^22^,^23^,^24^,^25^, and high-energy ball milling^13^. Each method has its own advantages to meet different experimental conditions and applications. Among all kind of methods, PVD and its ramifications are shown to be the most compatible with MEMS.

In 2003, Blobaum *et al.* demonstrated a sputtering method for deposition of CuO_x_/Al multilayer foils, and investigated the reaction path and growth kinetics in CuO_x_/Al thermite reaction. Their results showed CuO_x_/Al foils could react in a highly exothermic manner^26^,^27^. This work opened the perspective for application of PVD in thermite films preparation. Afterwards, Bahrami *et al.* prepared multilayered CuO/Al thermites in nanoscale and microscale using magnetron sputtering. After the comparison of the thermite reactions in different structures, the results confirmed that nanostructures made contribution to faster burning rate and higher energy release^28^. Zhu *et al.* obtained similar conclusion by comparing MoO_x_/Al multilayer nanofilms with different ratios of bilayer thicknesses^29^. This multilayer approach has the advantages of controllable ratio of reactants and compatibility with MEMS, but the fabrication process is expensive and requires high performance instrument. Moreover, nanoscale multilayer films with a certain thickness generally require dozens of modulation cycles, which is time consuming and discommodious. Recently, one-dimensional nanowires (NWs) were developed to prepare core/shell nanothermite, in which NWs were synthesized by thermally annealing metal film followed by deposition of Al shell^5^,^30^. Such core/shell structures can increase contact area and lower activation energy effectively. However, there are some concerns over this route: the length of NWs is not arbitrary; only a small fraction of metal oxide is included in NWs while most of them are still in the form of original film that appears in microscale. These limitations have a negative impact on the energy release and reaction rate^24^. In addition, just few types of nanothermite have been prepared by the aforementioned two approaches.

Inspired by these two approaches above, in order to produce well-defined nanothermites, a novel 3DOM method has been developed in our lab, in which, the 3DOM metal oxide framework is prepared using inverse template method followed by depositing a layer of Al film using magnetron sputtering^31^. The distinct 3DOM structure guarantees the compact combination of fuel and oxidizer in nanoscale and enlarges the effective contact area significantly. This approach is very easy for popularization and mass production. The feasibility of this method has been preliminarily verified in previous work through Fe_2_O_3_/Al system^31^,^32^, and the energy release is significantly larger than those previously reported from other structures^17^,^21^,^33^. Notably, most of metal oxides have been successfully formed into 3DOM structure^34^,^35^. Figure 1 shows the comparison of three different fabrication methods of nanothermite that are reviewed above. In this paper, Mn_2_O_3_ has been selected as an oxidant to prepare nanothermite, which is the first attempt in the literature. Through the investigation of different aluminizing time or molar ratio effects on the morphology and energy release, the reaction path of Mn_2_O_3_/Al nanothermite is widely discussed.

## Results and Discussion

### Phase Analysis

**Figure 2:**
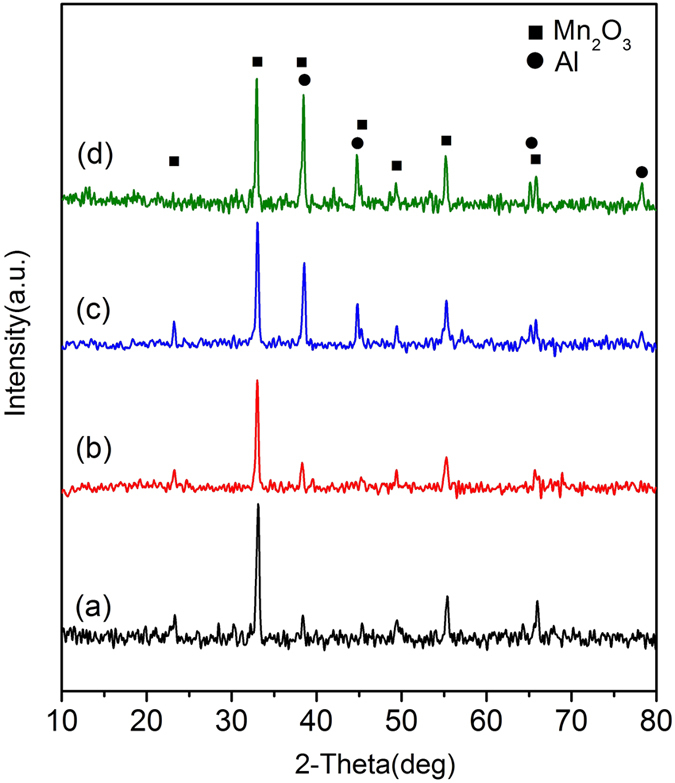
XRD patterns of (**a**) 3DOM Mn_2_O_3_ skeleton, (**b**) Mn_2_O_3_ nanoparticle, (**c**) 3DOM Mn_2_O_3_/Al membrane, (**d**) Mn_2_O_3_/Al nanoparticle composites.

The phase of the crystalline components is identified by Powder X-ray diffraction (XRD) characterization. As shown in Fig. 2a XRD pattern of 3DOM Mn_2_O_3_ skeleton exhibits a strong peak at 32.95°, and some weak peaks at 23.13°, 38.23° and 55.19°, just similar to the purchased Mn_2_O_3_ nanoparticle in Fig. 2b, corresponding to planes (222), (211), (400) and (440) of bixbyite α-Mn_2_O_3_(JCPDS 41–1442), respectively. Figure 2c shows XRD pattern of 3DOM Mn_2_O_3_/Al composite membrane. As expected, the diffraction peaks of Al are observed at 38.47°, 44.74° and 65.13°, corresponding to planes (111), (200), and (220) of Al. Simultaneously, the characteristic peaks of bixbyite Mn_2_O_3_ are kept in the same places without impure peaks, which reveals that phase transformation of Mn_2_O_3_ is avoided and the oxidation-reduction reaction between Al and Mn_2_O_3_ has not happened. These advantages are owing to the high-vacuum and the sustained low temperature during the aluminizing process. Over the Mn_2_O_3_/Al nanoparticle composites (NPC) sample, XRD pattern also indicates good crystallinities of Al and Mn_2_O_3_ after grinding with care.

### Morphology Characterization

**Figure 3:**
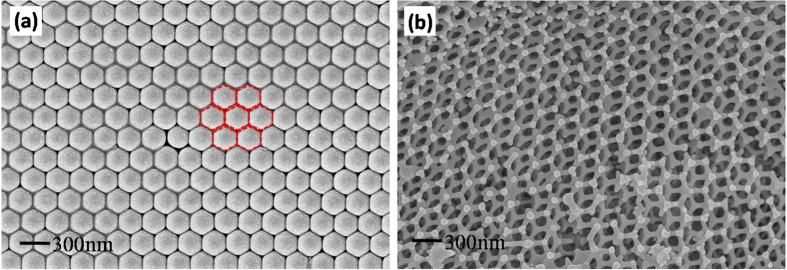
SEM images of (**a**) PS colloidal crystal template, (**b**) 3DOM Mn_2_O_3_ skeleton.

Scanning electron microscope (SEM) images of polystyrene (PS) colloidal crystal template (CCT) prepared by vertical deposition are shown in Fig. 3a. The monodispersed PS spheres are with bright surface and assembled into hexagonally-close packed structure which has been proved to be one of the most stable arrangements on thermodynamics^36^. Each sphere is surrounded by another six with uniform size and their planes oriented parallel to the surface of the microslide substrate, see red dash lines in Fig. 3a. The average diameter of the regular PS spheres is approximately 270 nm. Figure 3b shows the distinct 3DOM Mn_2_O_3_ structure, a high quality inverse replica of the template. Interconnected and ordered macroporous structure is observed after PS spheres are removed. A “honeycomb” structure is constituted by well-ordered air spheres and metal oxides walls in three dimensions with a thickness of 48 nm, and several layers within the skeleton are still visible in the surface image. The pore size is about 220 nm which corresponds to a pore shrinkage rate of approximately 18.5% compared with the original PS sphere. Some point defects and line defects are visible in the image. This is due to incomplete filling of the precursor and the transformation of the CCT during the calcination. Even so, we can consider that the integrity and consistency of the Mn_2_O_3_ skeleton are still maintained.

**Figure 4:**
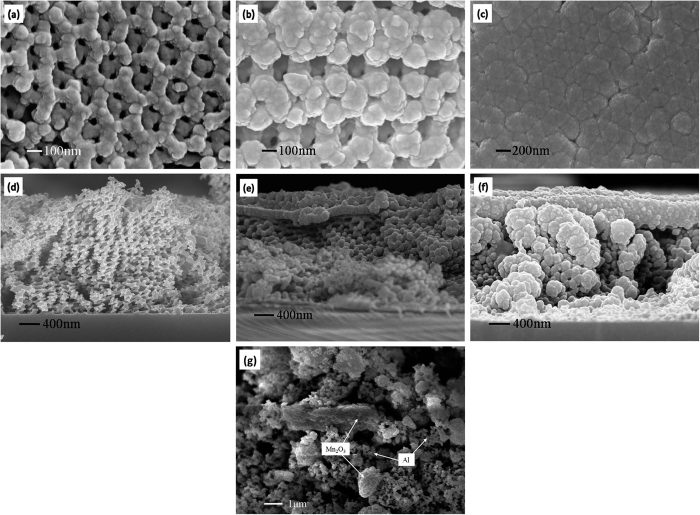
SEM images of Mn_2_O_3_/Al membrane with different aluminizing time of Al: (**a,d)** 10 min, (**b,e**) 20 min, (**c,f**) 30 min; (**a–c**) surface view and (**d–f**) cross-section view. (**g**) a typical Mn_2_O_3_/Al nanoparticle composite.

Figure 4 shows SEM images from Mn_2_O_3_/Al membrane with three different aluminizing times and a typical Mn_2_O_3_/Al nanoparticle composite. From surface view in Fig. 4a–c, one can see the rigid strength of the Mn_2_O_3_ skeleton is strong enough to support the deposited Al, and there is no fracture or collapse phenomenon. With aluminizing time increasing, the coated layer on the Mn_2_O_3_ wall is getting thicker, and the pore size is reduced. Figure 4a shows that wall thickness increases from the initial 48 nm to 86 nm because of deposition of Al, and increases further to 140 nm in Fig. 4b. When aluminizing time is increased to 30 min, only the wall trace can be seen dimly, and the pores are entirely filled. The same conclusion can be reached from the cross-section view of the samples in Fig. 4d–f, which reveal face-centered cubic (FCC) structure in three-dimensional view. The whole thickness of the composite membrane is about 2.6 μm.

**Figure 5:**
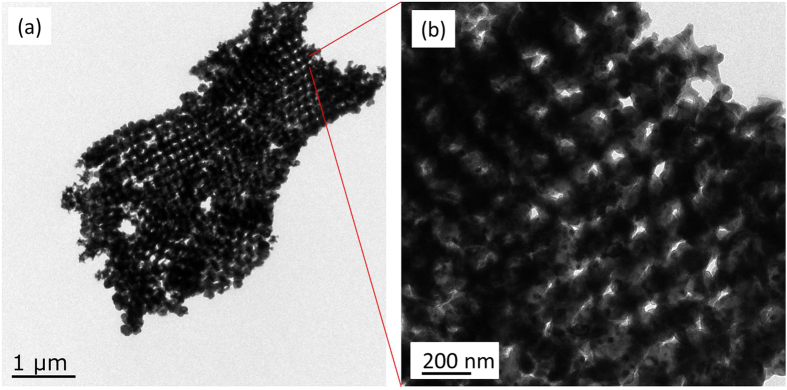
TEM images of Mn_2_O_3_/Al membrane with aluminizing time of 20 min.

For comparison, Fig. 4g shows the SEM image of Mn_2_O_3_/Al NPC. Mn_2_O_3_ particles are in the form of irregular morphology in nanoscales or macroscales, and the spherical Al particles are distributed among the Mn_2_O_3_ particles. One can see that Mn_2_O_3_ particles are severely agglomerated due to the high surface energy, and the dispersion homogeneity of Mn_2_O_3_/Al NPC is significantly lower than 3DOM Mn_2_O_3_/Al membrane. Furthermore, the cross-section view also has proved that Al is not only deposited on the surface, but presented in the interior of the Mn_2_O_3_ inverse template, see further discussion in the next section with X-ray photoelectron spectroscopy (XPS) results. In order to further exam the morphology of the Mn_2_O_3_/Al membrane, transmission electron microscope (TEM) images on cross section of Mn_2_O_3_/Al membrane with aluminizing time of 20 min are showed in Fig. 5. One can see Al is coated on 3DOM Mn_2_O_3_ skeleton to form a core/shell structure, and the nanothermite membrane can be viewed as an assembly of multiple nanothermite units, which are in agreement with the results obtained by SEM. Since Al is coated on Mn_2_O_3_ uniformly and connected with the skeleton closely both on the surface and inside the structure, one can expect the significantly enhanced effective contact area and improved reactivity.

### Elemental Analysis

**Figure 6:**
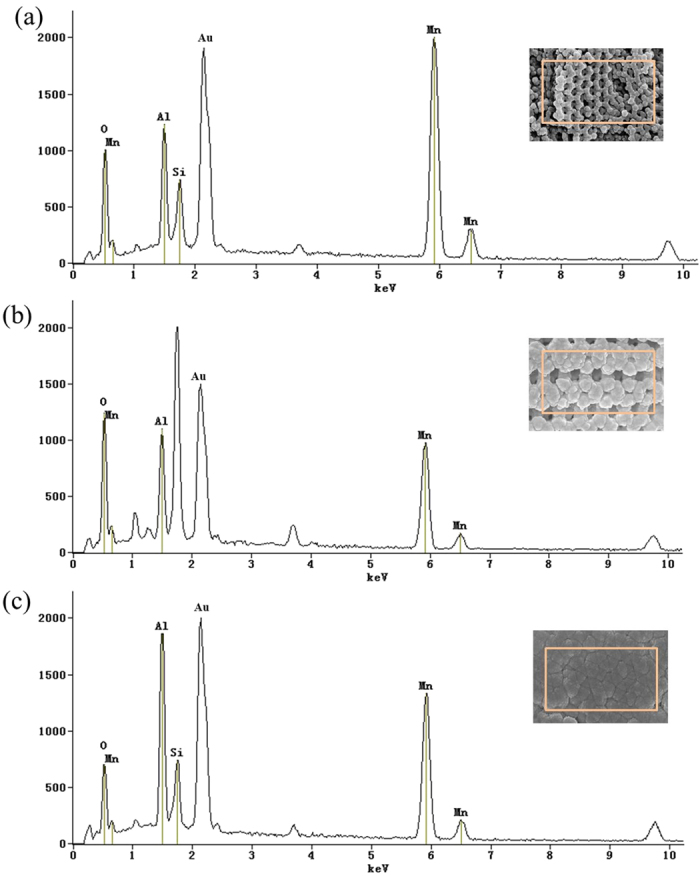
EDS spectrums of the Mn_2_O_3_/Al membrane with different aluminizing time. (**a**) 10 min, (**b**) 20 min, (**c**) 30 min.

Energy dispersive spectrum (EDS) is used to obtain a further insight into the aluminizing time on the influence of the elemental ratio. Figure 6 shows the EDS spectra of the Mn_2_O_3_/Al membranes with corresponding SEM images. Al and Mn peaks are observed in the spectra, accompanying with some impurity peaks that should be ignored, such as Au and Si. Au is from the layer deposited onto samples prior to the SEM measurement in order to increase the electric conductivity, while Si signal is from the quartz substrate. It is visualized that the percentages of Al peaks are gradually increased exactly as shown in SEM images, see EDS elemental mapping in Fig. S1 in supporting information.

The atomic percentages of the element (%) are obtained by integration of the peak areas, and molar ratio of Al to Mn_2_O_3_ is then calculated, respectively. Due to the presence of impurities, only Al and Mn are taken into account, see Table 1. The results show that the molar ratio of Al to Mn_2_O_3_ increases gradually with the increasing of aluminizing time, and they are linearly related because of the constant deposition rate of Al. Since the quantity analysis relies heavily upon the homogeneity of the samples, this is an indicative estimation in quantitation. One can see the molar ratio of Al to Mn_2_O_3_ is slightly larger than the stoichiometric value of the exothermic self-propagating reaction between Al and Mn_2_O_3_ when the aluminizing time is added to 30 min.

**Figure 7:**
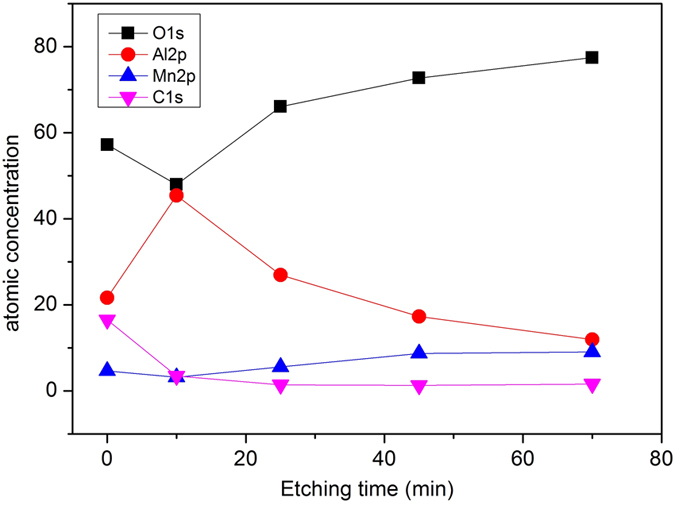
XPS depth profiling on atomic concentration within the Mn_2_O_3_/Al membrane as a function of Ar^+^ etching time.

In order to examine the elemental composition in the deeper layers within the nanothermite membrane, XPS depth profiling has been investigated with the aid of Ar^+^ sputtering on Mn_2_O_3_/Al membrane of aluminizing time of 30 min. The acceleration energy of ion beam is set to 3000 eV, corresponding to the etching rate of about 12 nm·min^−1^. A wide range survey, Mn2p and Al2p taken after each cycle of sputtering are shown in Figs S2–S4 in supporting information. Figure 7 shows calculated atomic percentage of the major elements displayed in XPS survey. The atomic percentage of Al goes to a maximum at an etching time of 10 min, which indicates that there is some excessive Al on the top of the membrane. With the increasing of etching time, the percentage of Al reduces while the percentage of Mn increases. However, even after 70 min etching, Al is still visible with a 12% atomic percentage in the membrane. One can easily conclude that Al has entered into the structure and coated the 3DOM Mn_2_O_3_ skeleton in three dimensions, which is also confirmed by TEM examination, see Fig. 5.

### Energy release

The self-propagating reaction between Al and Mn_2_O_3_ would happen under the stimulus of flame, laser pulse, current, heating, etc. The reaction equation is described in the following equation (1),


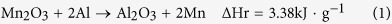


**Figure 8:**
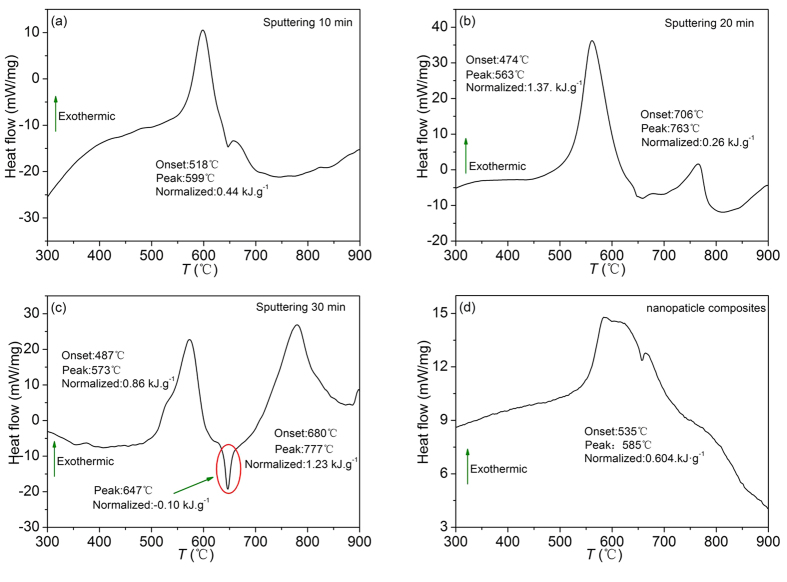
DSC curves of Mn_2_O_3_/Al membrane obtained in a temperature range from 200 to 900 °C with a heating rate of 20 °C·min^−1^ under a 30.0 mL·min^−1^ N_2_ flow with different aluminizing times. (**a**) 10 min, (**b**) 20 min, (**c**) 30 min, and (**d**) DSC curve of Mn_2_O_3_/Al nanoparticle composites.

The thermite reaction of Mn_2_O_3_/Al nanothermite membrane is characterized with differential scanning calorimetry (DSC). The DSC curves from Mn_2_O_3_/Al nanothermite membranes obtained with different aluminizing times are shown in Fig. 8. Only a small exothermic peak is arisen from the sample with aluminizing for 10 min in Fig. 8a, which indicates that Al is far from enough, in agreement with the molar ratio of Al to Mn_2_O_3_ is only 0.73 calculated by EDS. When the aluminizing time is added to 20 min, see Fig. 8b, there are two major exotherms, but most of energy is released in the first exotherm. The second exothermic reaction starts after the melting point of Al at 660 °C. In addition, a weak endothermic peak is also observed. This is because the product of thermite reaction at interface prevent Al to react completely^21^. As shown in Fig. 8c, the larger endothermic peak reveals that there is excessive Al after the first exothermic reaction. The heat absorption capacity is 0.10 kJ∙g^−1^. All these peaks are with sharp profile, which suggests the energy release in a short period of time. Therefore, it implies that the thermite reaction between Al and Mn_2_O_3_ can be divided into two steps. First reaction is based on solid-solid diffusion mechanism, and second reaction triggers by liquid-solid diffusion mechanism after Al is melted, which is similar to NiO/Al system that has been reported before^37^. In comparison, the DSC curve of Mn_2_O_3_/Al NPC under the same test conditions is shown in Fig. 8d. The profile of exothermic peak from NPC is similar to Fig. 8a, and the output of energy is just a little higher than Mn_2_O_3_/Al membrane at aluminizing time of 10 min, which reveals that reactants are not able to react completely, although Al and Mn_2_O_3_ are mixed in the stoichiometric ratio.

In order to compare the influence of aluminizing time on the DSC results, all the experimental data are collectively listed in Table 2. It shows that total energy release has achieved its maximum, 2.09 kJ·g^−1^, when the aluminizing time is 30 min. Its first energy release is not the largest, but the second one is five times larger than that from the sample with 20 min aluminizing. This is owing to the excessive Al on the interface. On the other hand, Al is insufficient in the sample with aluminizing time of 20 min. We draw the conclusions that the first exothermic reaction is mainly controlled by the coverage of the fuel. When it’s too thick, products cumulated on the interface may prevent the reaction to progress further, but the reaction is resumed after the residual Al is melted. Therefore, the total energy release is determined by the Al layer thickness deposited on the Mn_2_O_3_ skeleton. In addition, the maximum energy release is still lower than the theoretical value of 3.38 kJ·g^−1^, which may be attributed to the unavoidable oxidation of Al film during storage, and the fact that Mn_2_O_3_/Al membrane is not in stoichiometric ratio. Despite all this, the energy release is still considerably high, and the first onset temperature is as low as 480 °C, which is approximately 50 °C lower than Mn_2_O_3_/Al NPC. These characteristics make the Mn_2_O_3_/Al nanothermite membrane a remarkable nanoenergetic material.

**Figure 9:**
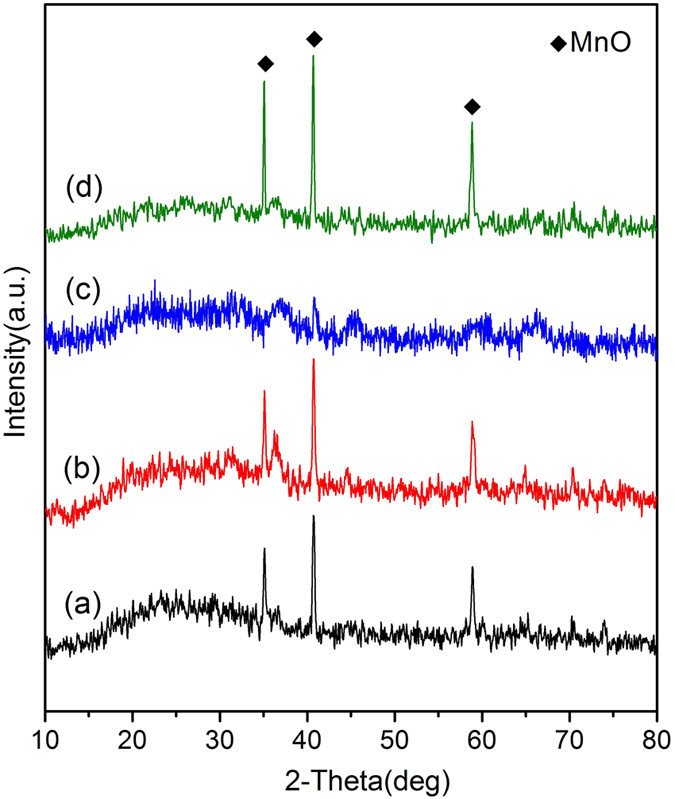
Corresponding XRD patterns of Mn_2_O_3_/Al nanothermite membranes and Mn_2_O_3_/Al nanoparticle composites after DSC test: (**a**) 10 min, (**b**) 20 min, (**c**) 30 min, and (**d**) Mn_2_O_3_/Al nanoparticle composites.

Figure 9 shows the corresponding XRD patterns of Mn_2_O_3_/Al nanothermite membranes and Mn_2_O_3_/Al NPC after DSC test. From the sample with aluminizing time of 10 min, in Fig. 9a, MnO (JCPDS 75-0626) peaks are observed without peaks from Al and Mn_2_O_3_. This means that Al has reacted with Mn_2_O_3_ unreservedly during heating process, and Al is insufficient to reduce Mn_2_O_3_ thoroughly. The oxidation–reduction reaction is expressed in the following equation (2):


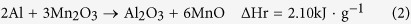


Similar pattern is obtained from the samples of Mn_2_O_3_/Al membrane with 20 min aluminizing, in Fig. 9b, and Mn_2_O_3_/Al NPC, in Fig. 9d. It is worth noting that the expected Al_2_O_3_ and Mn peaks are not presented. That is probably due to the product of Al_2_O_3_ and Mn are amorphous or nanocrystalline^3^,^27^,^38^. The XRD pattern from the sample with aluminizing time of 30 min, in Fig. 9c, doesn’t exhibit any characteristic peaks of MnO, which reveals that MnO has been reduced completely.

### Laser Ignition Test

**Figure 10:**
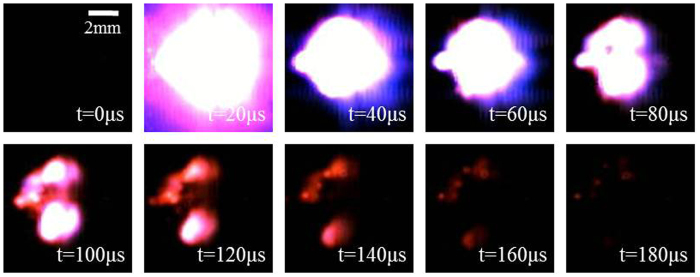
High speed camera observation of laser ignition test for Mn_2_O_3_/Al nanothermite membrane with 30 min aluminizing time. The incident laser energy is 74 mJ per pulse.

The ignition performance of 3DOM Mn_2_O_3_/Al nanothermite membrane has been tested with the sample of 30 min aluminizing. We assumed that one frame prior to the point of light appearing to be the initial time, and a sequence of test results with an interval of 20 μs between adjacent images are shown in Fig. 10. After a laser pulse struck upon Mn_2_O_3_/Al nanothermite membrane, a bright flash of white light surrounded by amaranthine glow burst out from the substrate at 20 μs, which indicated that the Mn_2_O_3_/Al membrane was ignited successfully. The whole ignition process lasted around 180 μs, and the maximum height of the flame was 8 mm at 20 μs. The high speed camera observations of ignition with other aluminizing time are shown in Fig. S5. As aluminizing time increased (from 10 minutes to 20 minutes, then to 30 minutes), the ignition duration also increased (100 μs, 140 μs, and 180 μs, respectively). The height of the flame gets higher and sparks became stronger. It can be concluded that the reaction between Al and Mn_2_O_3_ releases more energy along with the increasing of aluminizing time, which is consistent with the DSC results. It can be definitely concluded that a thermite reaction between Al and Mn_2_O_3_ occurred under the stimulus of laser pulse.

## Summary

In this work, Mn_2_O_3_ is selected to combine with Al to achieve nanothermite of Mn_2_O_3_/Al. The 3DOM Mn_2_O_3_/Al nanothermite membrane is prepared via a simple and feasible procedure: vertical deposition of CCT, inverse template method for 3DOM Mn_2_O_3_ skeleton and magnetron sputtering for Mn_2_O_3_/Al nanothermite membrane. SEM results reveal that the Mn_2_O_3_ skeleton is highly ordered with interconnected and uniform macroporous structure, and Al is deposited uniformly onto the 3DOM Mn_2_O_3_ skeleton in order to form nanothermite membrane throughout the structure. With the increasing of aluminizing time, the Al layers get thickened. An optimized total energy release from nanothermite membrane of 2.09 kJ·g^−1^ with the onset temperature of around 480 °C is achieved when the aluminizing time is increased to 30 min. In addition, laser ignition test results show a bright flash of light lasting up to 180 μs. The 3DOM structural nanothermites possess the advantages of high energy density, large contact area, and short mass transfer distance that insure large energy release. Moreover, this method develops a new avenue to prepare various nanothermite membranes compatible with MEMS.

## Methods

### Fabrication of the 3DOM Mn_2_O_3_ skeleton

The PS spheres were synthesized by emulsifier-free polymerization technology in our laboratory^31^. The prepared PS spheres were assembled into a 3DOM CCT via vertical deposition method. In a typical procedure, an absolutely clean microslide substrate was vertically inserted into a spheres suspension (1.5 vol %), then, dried in the oven at a constant temperature of 50 °C for 5 days. The number of CCT layers can be controlled by adjusting concentration of the PS suspension. Driven by a substrate–solution–air interface capillary action, PS spheres were deposited on the substrate gradually and in an orderly manner. 3DOM CCT was obtained when the solvent has evaporated completely. After that, the void space among the CCT was infiltrated with precursor and then removed to fabricate 3DOM metal oxides membrane. The selection of precursor is crucial. In this paper, Mn(Ac)_2_ 4H_2_O(2.0 mol·L^−1^) was selected as the Mn source, which was dissolved in a mixture solution of methanol and ethylene glycol (volumetric ratio = 2/3). Simultaneously, moderate Polyvinyl Pyrrolidone (PVP) was added to increase the viscosity of the mixed solution. The influences of PVP on morphology are shown in Fig. S6 in the Supporting Information. After 12 hours of magnetic stirring, a magenta sol was formed and CCT was dipped into the prepared precursor vertically for 5 min. The residual liquid on substrate surface must be wiped off immediately once the template was lifted out of the precursor solution. The template filled with liquid precursor was then placed in an oven kept at 60 °C for 4 hours to solidify the structure. At last, in order to remove the organic components the sample was calcined in a furnace with a heating rate of 1 °C∙min^−1^ and kept at 500 °C for 1 hour in an open atmosphere.

### Fabrication of the 3DOM Mn_2_O_3_/Al nanothermite membrane

After the 3DOM Mn_2_O_3_ membrane was formed, Al was deposited onto the 3DOM Mn_2_O_3_ skeleton by magnetron sputtering to form core/shell structures with the 3DOM Mn_2_O_3_ as the core and Al as the shell. Prior to the Al deposition, the 3DOM Mn_2_O_3_ skeleton was rinsed by a small amount of anhydrous ethanol and dried under N_2_. All the sputtering process was under the vacuum condition of 5 × 10^−3^ Pa, the DC voltage 150 V, and ultrahigh purity Ar as functional gas with the flux of 30 sccm. On account of the high-vacuum and sustained low temperature, the oxidation of Al or the reaction of Mn_2_O_3_ and Al were mostly prevented during this process. In order to explore the influence of Al film thickness on the energy release from Mn_2_O_3_/Al nanothermite membrane, various amounts of Al was deposited by varying the thickness of Al membrane, through controlling the aluminizing time. Three different aluminizing times were designed: 10 min, 20 min and 30 min, respectively.

For comparison purposes, Mn_2_O_3_/Al NPC were also prepared via classic physical mixing. Aluminum particles with an average size of 50 nm were obtained from Aladdin Industrial Corporation (Shanghai, China), and Mn_2_O_3_ were purchased from Dk Nano Technology Corporation (Beijing, China). Stoichiometric mixtures of the reactants were separately mixed in hexane for 5 min in an ultrasonic bath and then added to one beaker for continuing ultrasonic dispersion for 30 min. The suspension was then moved to a vacuum oven kept at 70 °C under 1.5 × 10^4^ Pa in order to obtain Mn_2_O_3_/Al NPC.

### Characterizations of samples

The morphology were characterized by a field-emission scanning electron microscope (FE-SEM) (Hitachi, S-4800) and a TEM (JEOl, JEM-2100). Elemental analysis of the 3DOM Mn_2_O_3_/Al nanothermite membrane was carried out using EDS. XPS were taken using an ESCALAB250Xi XPS instrument (Thermo Scientific). A monochromatic Al Ka x-ray source (1486.6 eV) was used with a spot size of 400 nm diameter. A pass energy of 100 eV and step size of 0.5 eV were used for survey spectra, and a pass energy of 20 eV and step size of 0.1 eV were used for high resolution spectra. Powder XRD (Bruker, D8Advance) was used to analyze the component of the material at each stage. Prior to the measurement, all the samples were striped from the microslide and attached onto a piece of flat quartz. In addition, the exothermic reaction of the 3DOM Mn_2_O_3_/Al nanothermite membrane was performed by DSC (METTLER TOLEDO, DSC 1) under N_2_ atmosphere in a temperature range of 200 to 900 °C with a flow rate of 30 mL·min^−1^, and the heating rate was set to 20 °C·min^−1^. A pulsed laser (Beamtech, DAWA-350) was used to investigate the ignition performance of the 3DOM Mn_2_O_3_/Al nanothermite membrane. The laser was set to wavelength 1064 nm, pulse width 6 ns, beam diameter 700 μm, repetition rate 10 Hz, and the incident energy was 74 mJ per pulse. A piece of glass substrate with Mn_2_O_3_/Al nanothermite membrane on it was placed at the focal point of the laser and a high speed camera was placed at a position perpendicular to the laser direction to observe the ignition process. The whole ignition process was recorded by a high speed camera (Redlake Motion Xtra, HG-100 K), and the data acquisition frequency was set as 50000 frames per second (fps).

## Additional Information

**How to cite this article**: Zheng, G. *et al.* Three-dimensionally Ordered Macroporous Structure Enabled Nanothermite Membrane of Mn_2_O_3_/Al. *Sci. Rep.* **6**, 22588; doi: 10.1038/srep22588 (2016).

## Supplementary Material

Supplementary Information

## Figures and Tables

**Figure 1:**
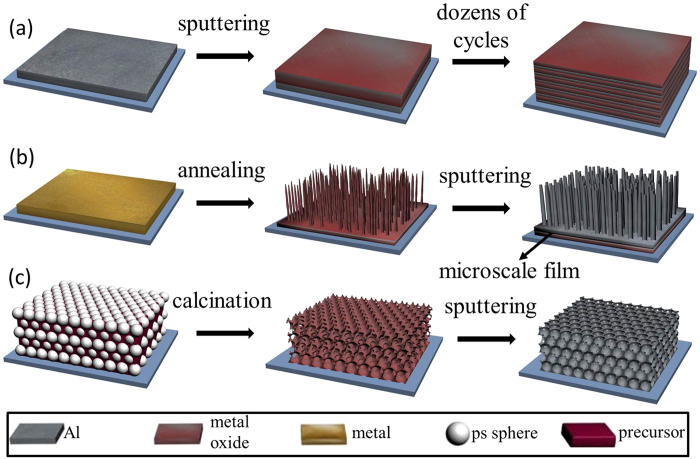
Comparison of three different fabrication methods of nanothermite: (**a**) multilayer films, (**b**) NWs core/shell structure, (**c**) 3DOM nanothermite membrane.

**Table 1:** Elemental composition of the products with different aluminizing time.

aluminizing time (min)	Atomic percentage of the elements (%)		Molar ratio of Al to Mn_2_O_3_
	Al	Mn	
10	26.74	73.26	0.73
20	45.43	54.57	1.66
30	55.75	44.25	2.52

**Table 2:** DSC results analysis of Mn_2_O_3_/Al nanothermite membrane with different aluminizing time.

Aluminizing time (min)	First onset temperature (°C)	First energy release (kJ·g^−1^)	Second onset temperature (°C)	Second energy release (kJ·g^−1^)	Total energy release (kJ·g^−1^)
10	518	0.44	–	–	0.44
20	474	1.37	706	0.26	1.63
30	487	0.86	680	1.23	2.09
