# Supplementary material for: Three-dimensionally Ordered Macroporous Structure Enabled Nanothermite Membrane of Mn2O3/Al
Source: Sci Rep. 2016 Mar 3;6:22588. doi: 10.1038/srep22588 (PMC4776280; doi:10.1038/srep22588)
Supplement: Supplementary Information [file srep22588-s1.pdf]

Supporting Information

Three-dimensionally Ordered Macroporous Structure Enabled Nanothermite Membrane of Mn_2O_3/Al

*Guoqiang Zheng,[†] Wenchao Zhang,[†,] * Ruiqi Shen,[†] Jiahai Ye,[†] Zhichun Qin,[†] and Yimin Chao[‡,] **

[†]School of Chemical Engineering, Nanjing University of Science and Technology, Nanjing 210094, China

[‡]School of Chemistry, University of East Anglia, Norwich NR4 7TJ, United Kingdom

1. SEM image of 3DOM Mn_2O_3/Al nanothermite membrane and corresponding SEM-EDS elemental mapping

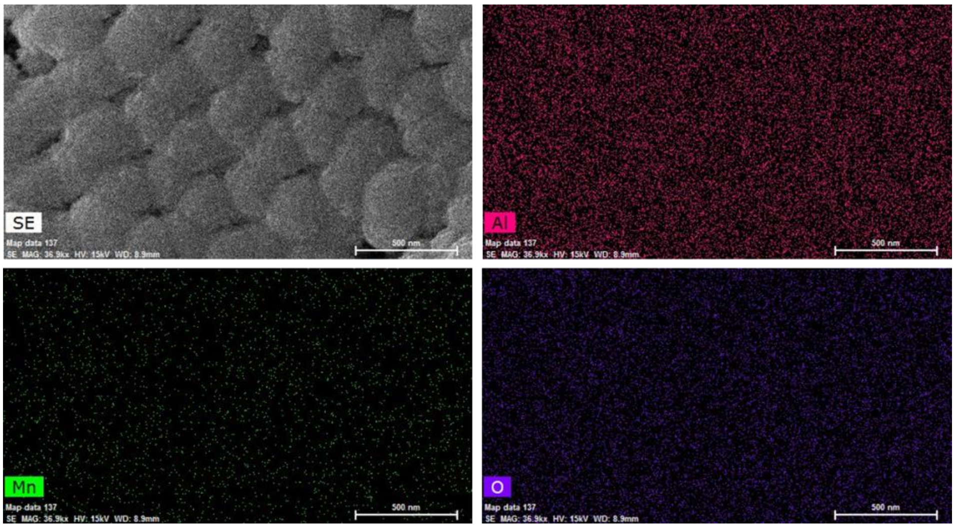

Figure S1. SEM image of 3DOM Mn2O3/Al nanothermite membrane at aluminizing time of 30 min and corresponding SEM-EDS elemental mapping of Al, Mn and O.

2. XPS spectra

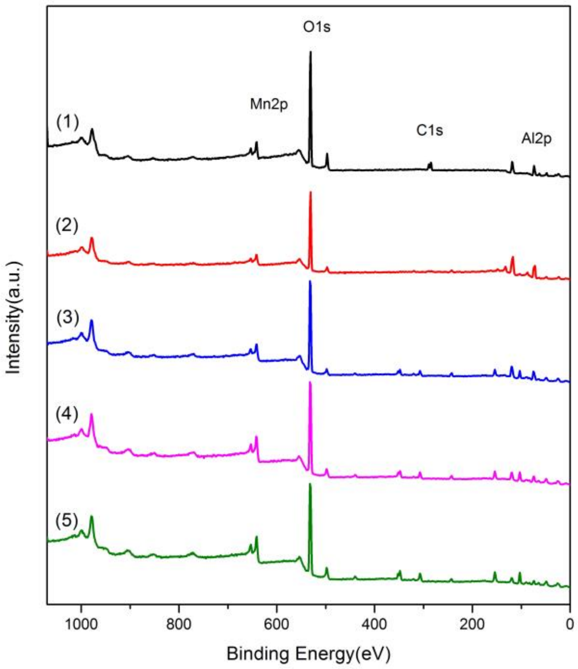

Figure S2. Survey spectra after various etching times: (1) 0 min; (2) 10 min; (3) 25min; (4) 45 min; (5) 70 min.

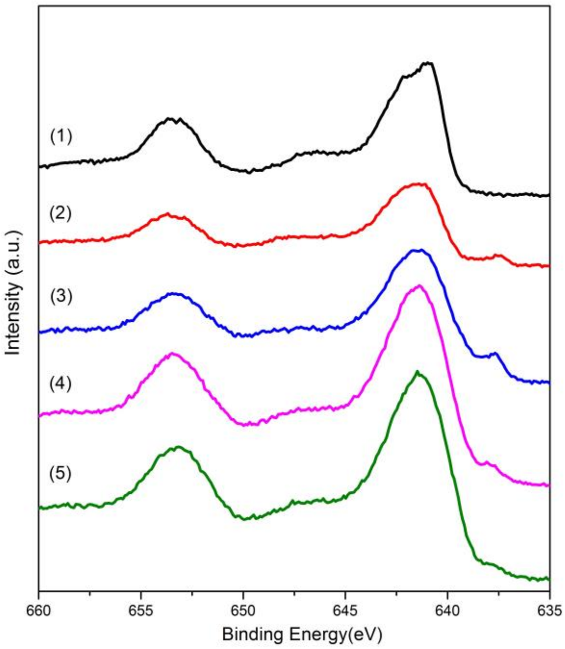

Figure S3. Mn2p spectra after various etching times: (1) 0 min; (2) 10 min; (3) 25min; (4) 45 min; (5) 70 min.

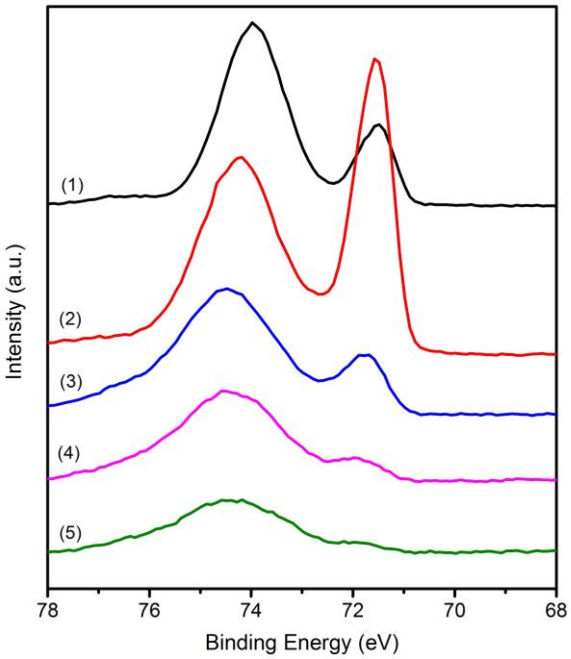

Figure S4. Al2p spectra after various etching times: (1) 0 min; (2) 10 min; (3) 25min; (4) 45 min; (5) 70 min.

3. High speed camera observation of laser ignition test for different aluminizing time.

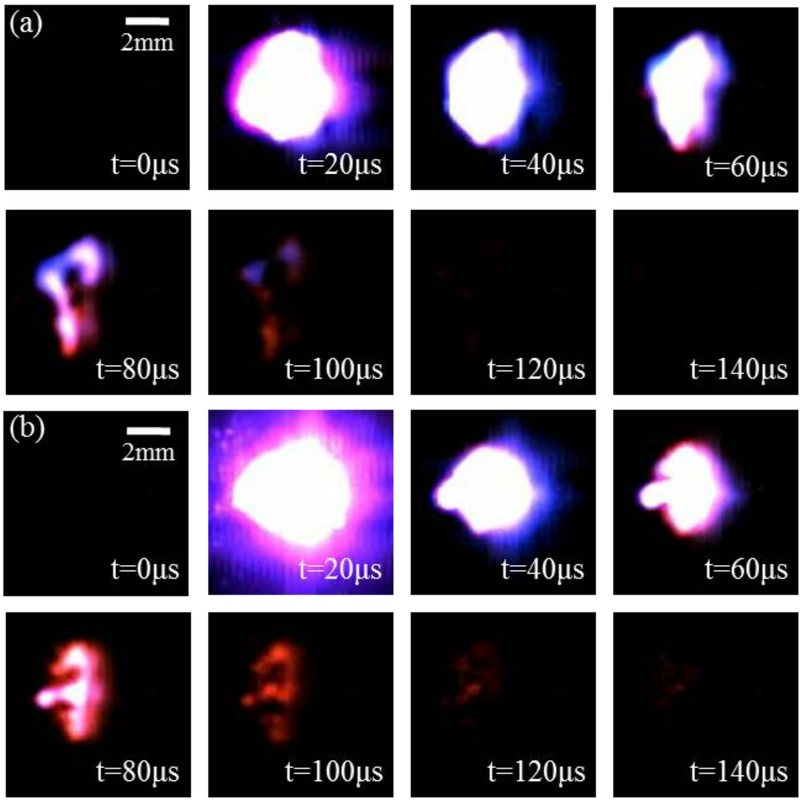

Figure S5.High speed camera observations of laser ignition test for different aluminizing time: (a) 10 min, (b) 20 min. The incident laser energy is 74 mJ per pulse

4. SEM images of 3DOM Mn_2O_3 skeleton with different amounts of PVP, which is dissolved in 20 ml mixture solution of methanol and ethylene glycol (volumetric ratio=2/3).

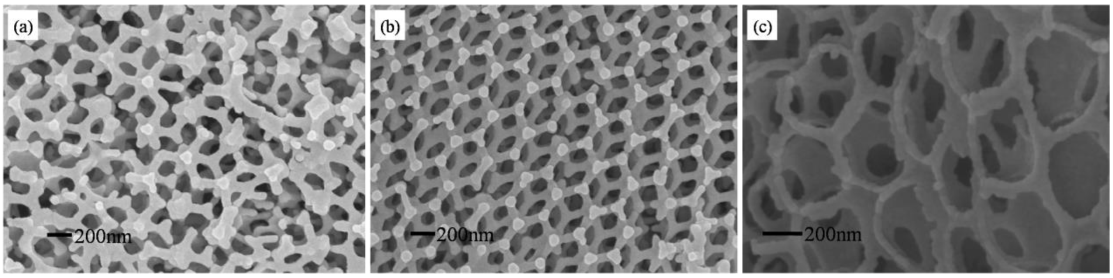

Figure S6. SEM images of 3DOM Mn2O3 skeleton with different amounts of PVP. (a) 0.2 g, (b) 1 g, (c) 2 g
